# Effects of Urbanization Expansion on Landscape Pattern and Region Ecological Risk in Chinese Coastal City: A Case Study of Yantai City

**DOI:** 10.1155/2014/821781

**Published:** 2014-04-10

**Authors:** Di Zhou, Ping Shi, Xiaoqing Wu, Jinwei Ma, Junbao Yu

**Affiliations:** ^1^Key Laboratory of Coastal Environment Processes and Ecological Remediation and Shandong Provincial Key Laboratory of Coastal Zone Environmental Processes, YICCAS, Yantai Institute of Coastal Zone Research, Chinese Academy of Sciences, Yantai 264003, China; ^2^University of Chinese Academy of Sciences, Beijing 100049, China; ^3^Linqu First Middle School Shandong, Linqu 262600, China

## Abstract

Applied with remote sensing, GIS, and mathematical statistics, the spatial-temporal evolution characteristics of urbanization expansion of Yantai city from 1974 to 2009 was studied. Based on landscape pattern metrics and ecological risk index, the landscape ecological risk from the landscape pattern dynamics was evaluated. The results showed that the area of urban land increased by 189.77 km^2^ with average expansion area of 5.42 km^2^ y^−1^ from 1974 to 2009. The urbanization intensity index during 2004–2009 was 3.92 times of that during 1974–1990. The land use types of urban land and farmland changed greatly. The changes of landscape pattern metrics for land use patterns indicated that the intensity of human activities had strengthened gradually in study period. The landscape ecological risk pattern of Yantai city shaped half-round rings along the coastline. The ecological risk index decreased with increase of the distance to the coastline. The ratio of high ecological risk to subhigh ecological risk zones in 2009 was 2.23 times of that in 1990. The significant linear relationship of urbanization intensity index and regional ecological risk indicated that the anthropological economic activities were decisive factors for sustainable development of costal ecological environment.

## 1. Introduction

It is common that the urbanization expansion is rapid in the world, especially in the developing countries where there are about 70% of the world's largest cities [1–3]. The urbanization has boosted rapidly in China since the reform and opening-up in the 1980s. The urbanized area in Yangtze River Delta region increased exponentially during 1979–2008 [4]. According to the report of the National Bureau of Statistics in 2012, the urbanization ratio of China has increased by 1.35% per year from 2002 to 2011. By the end of 2011, the total urban population has been about 690.69 million people and the urbanization ratio was about 51.27%. The human development report 2013 of the United Nations Development Programme predicted that the urban population of China will be 1000 million people and the urbanization ratio will be 70% in 2030. Up to now, more than 40% of population and more than 70% of cities are widely distributed in China's coastal areas [5] where many coastal economic zones are set up and have become the preferred destination for millions of internal migrants and overseas investors [6]. Some studies indicated that the urbanization ratio will increase to 60% in 2020 in the eastern coastal provinces and cities of China [7].

The rapid development of urbanization expansion and heavy burden of human activities have impaired ecosystem functions in coastal areas [8–12]. From the view of landscape ecology, urbanization is the process in which land use/cover landscape changes from natural landscape which is mainly made of water, soil, and vegetation to manmade landscape which is mainly composed of cement, asphalt, chemical materials, and metal [13, 14]. Meanwhile, the change of land use/cover landscape pattern could be interpreted as the changes of patch shape, area, quality, and spatial combination [15, 16]. Rapid urbanization has been companied by more drastic changes of land use types, disorderly landscape layout, and fragile ecological environment [14, 16, 17]. Therefore, the problems of landscape pattern change and region ecological risk under urbanization expansion in China have drawn the increasing attention of the ecologists [4–10]. The technologies of RS and GIS have become important methods to identify the potential eco-environmental influences and guide urban planning management [3, 15, 18–22]. The previous researches in China mainly focused on large cities distributed in the Yellow River [23], the Yangtze River [24, 25], Pearl River Delta [9, 26], and the Ring-Bohai Region [27–29]. Few studies have focused on landscape pattern and region ecological risk under urbanization expansion in the small and medium-sized coastal cities [30–32]. In the study, Yantai city, which was one of the 14 coastal open economy cities in China, was selected to study the impacts of urbanization expansion on the landscape pattern change and regional ecological risk for the small and medium-sized coastal cities. The objectives of the present study were to reveal (1) dynamic changes and spatial characteristics of landscape pattern and the regional ecological risk and (2) the relationship between urbanization intensity index and regional ecological risk based on the analysis of urbanization expansion in Yantai city.

## 2. Materials and Methods

### 2.1. Description of the Study Area

**Figure 1:**
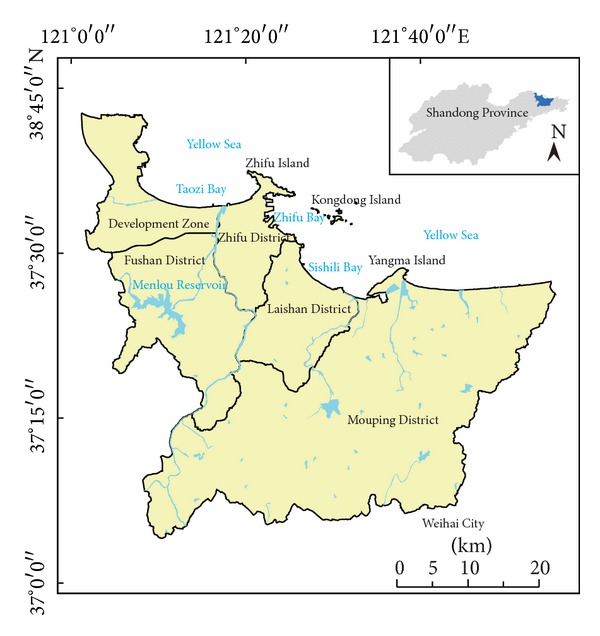
Location map of the study area.

Yantai city (119°34′–121°57′E, 36°16′–38°23′N) is located at the northeast tip of Shandong Peninsula bordering on the Bohai Sea and the Yellow Sea (Figure 1). It covers an area of 13746.50 km^2^ with the coastline of 909.00 km. The mountain, mound, plain, and the marsh land are about 36.6%, 39.7%, 20.8%, and 2.9% of total area, respectively. Average annual precipitation is 651.90 mm and mean annual temperature is 11.8°C. The total population was 6.96 million in 2010 based on the 6th population census data of China. The gross domestic product (GDP) was about 490.68 billion Chinese YUAN according to the government work report of Yantai City in 2011. With the improvement of comprehensive competitiveness, Yantai city has been an important economic zone of the Ring-Bohai Region.

Five districts of Yantai city, that is, Zhifu District, Laishan District, Mouping District, Fushan District, and Economic and Technological Development Zone (Development Zone), were selected for the study (Figure 1). The study area (121°15′–121°56′E, 37°04′–37°38′N) covers an area of 2721.04 km^2^ and the total population is about 2.22 million in 2010. With rapid economic development and enormous population growth, large area agricultural land has been occupied by nonagricultural lands and urban landscape pattern resulting from urbanization expansion in the past years.

### 2.2. Data Sources and Processing

#### 2.2.1. Remote Sensing Image Interpretation

The spatial data was obtained from five-period remote sensing images June 2, 1990 (TM), September 20, 1995 (TM), June 12, 2000 (ETM), November 9, 2004 (TM), and July 15, 2009 (TM). In addition, administrative division maps, traffic map, topographic map (1 : 100,000) in 1974, topographic map (1 : 50,000) in 2000, and land use planning of Yantai city were used for secondary data. The five period remote sensing images of 1990, 1995, 2000, 2004, and 2009 were used to study the spatial-temporal evolution characteristics of urbanization expansion in Yantai city. Three period images of 1990, 2000, and 2009 were applied for the analysis of the land-use landscape pattern characteristics and region ecological risk.

The topographic maps of Yantai city, geometric correction of Landsat TM remote sensing images had been conducted by using ArcGIS 9.3. The error was controlled within 0.5 pixels. All images were classified by combing supervised classification with visual interpretation and the interpretation results were resampled in spatial resolution (30 m).

According to the Chinese national standard of Status of Land Use Classification (GB/T 21020-2007), the land use types of the study area were divided into urban land, farmland, orchard, forestland, grassland, water body, other construction land (industrial land, mine land, and port land), and other object land (unused land, barren land). Based on land use interpretation results which were corrected by field survey, the classification error rate was lower than 5% of the land use actual change.

#### 2.2.2. Urbanization Expansion

The urbanization intensity index (*I*_ue_) was used to analyze the urbanization expansion intensity from 1974 to 2009. The equation was as follows:

(1) $$\begin{array}{c} I_{\text{ue}}=\frac{\Delta U_{i}\times 100}{\text{TLA}\times \Delta t}, \end{array}$$

where *I*_ue_ is urbanization intensity index, Δ*U*_*i*_ is expansion area of urban land during a certain period, TLA is the total area of the research area, and Δ*t* is time span of a certain duration.

#### 2.2.3. Landscape Pattern Metrics

The six landscape metrics of Largest Patch Index (LPI), Fractal Dimension Index (FD), Shannon's Diversity Index (SHDI), Shannon's Evenness Index (SHEI), Dominance Index (Dominance), and Contagion Index (CONTAG) were selected for landscape pattern analysis. As a measure of landscape heterogeneity, SHDI is especially sensitive to the nonbalanced distribution of all patch types in the landscape. SHEI is applied to indicate the diversity of different landscapes or a certain landscape in different periods, which results in maximum evenness. As such, evenness could be the complement of dominance. Contagion Index is related to edge density and can measure the clumping trends of the patches. The higher the CONTAG is, the more homogeneous and contiguous the spatial pattern is. The Fragstats 3.3 was adopted to calculate and analyze landscape pattern metrics. Expressions and ecological significance of metrics in the present study are given in Table 1 [33–38].

#### 2.2.4. Ecological Risk Assessment

The ecological risk index (ERI) [39] (2) was adopted to assess the spatial difference of comprehensive ecological risk in the various land use types under urbanization expansion:

(2) $$\begin{array}{c} \text{ERI}=\overset{N}{\underset{i=1}{\sum }}\frac{A_{i}}{A}w_{i}, \end{array}$$

where ERI is ecological risk index; *i* is land use type;  *A*_*i*_ is area of land *i*; *A* is the total area of research area; *w*_*i*_ which is the comprehensive ecological risk intensity reflected by *i* is determined by the method of the analytic hierarchy process (AHP) for each land use type. The values of *w*_*i*_ for urban land, farmland, orchard, forestland, grassland, water body, other construction land, and other object land are 0.29, 0.13, 0.11, 0.03, 0.05, 0.04, 0.20, and 0.07, respectively.

Based on the geostatistical analysis module of ArcGIS 9.3, the studied area was divided into 370 sampling grid units with the size of 3 km × 3 km to establish the evaluation unit of ecological risk after equidistant partition. The ecological risk index values were calculated in each grid units. The spherical model method was applied to control test fitting results. Together with ordinary Kriging method for data interpolation, the regional ecological risks in 1990–2009 were evaluated. Finally, with calculating results of the urbanization intensity index and variation of ecological risk of the evaluation unit, the regression analysis method was used for the quantitative relationship between urban spatial expansion and regional ecological risk.

## 3. Results and Discussion

### 3.1. The Spatial-Temporal Evolution Characteristics of Urbanization Expansion

**Figure 2:**
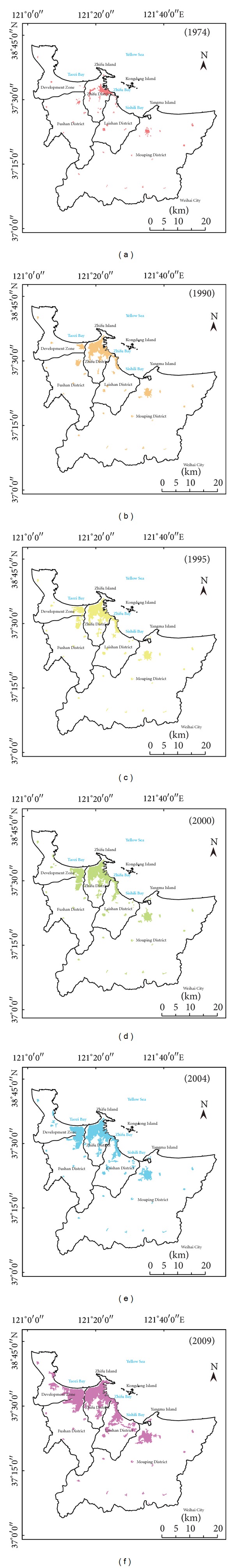
Spatial distribution of urban land in the study area.

**Figure 3:**
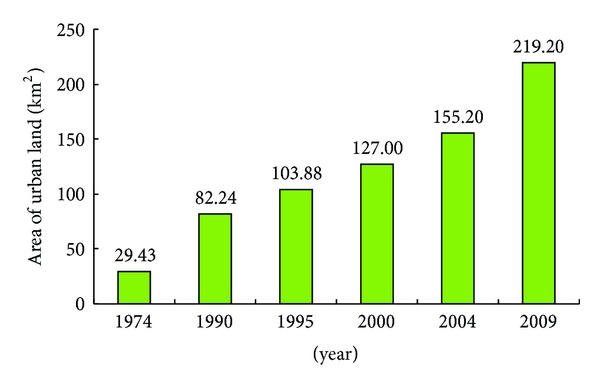
Area of urban land during the period of 1974–2009.

The urbanization process in Yantai city speeded up sharply since the reform and opening-up policy (1980s) (Figures 2 and 3). The area of urban land occupied 29.43 km^2^ and only distributed in the center of Zhifu District and Muping District in 1974. The urban area increased 52.81 km^2^ from 29.43 km^2^ in 1974 to 82.24 km^2^ in 1990. Meanwhile, as an important coastal city in China, the port industry developed rapidly, such as the construction of Yantai Port in Zhifu District. Therefore, urban land not only expanded to the northern and western part of Zhifu District, but also appeared in the connection between Zhifu District and Development Zone (Figure 2). The rapid development of urbanization expansion occurred in period of 1990–2009. The area of urban land expanded to 127.00 km^2^ in 2000 and 219.20 km^2^ in 2009, respectively (Figure 3). Restricted by geographical limit, the distribution of urban land in Zhifu District began to spread to Zhifu Island (northern part of Zhifu District) and Huangwu Town (southern part of Zhifu District) after 1990. Because of the migration of Yantai municipal government from Zhifu District to Laishan District since 2000, the Laishan District has become a new hot spot of urban land expansion. In addition, the study area had been well served with traffic facilities including Laishan Airport, Yantai-Weihai highway, Tongjiang-Sanya highway, 010 National Highway, and 204 National Highway. The Shanhai Road which was constructed in 2003 has connected Fushan District, Zhifu District, and Laishan District. Meanwhile, the port industry (such as Bajiao Port in Development Zone) and a series of coastal leisure planning related coastal tourism economy developed quickly in the study area. Therefore, urban land in Yantai city expanded dramatically from coastline to inland region during 2000 and 2009.

The area of urban land of Yan city increased by 189.77 km^2^ with annual expansion area of 5.42 km^2^ and Urbanization Intensity Index of 0.20 during 1974–2009 (Table 2). The urbanization process during studied period can be divided into three stages, that is, initial stage (1974–1990), development stage (1990–2000), and rapid expansion stage (2000–2009) (Table 2). The urbanization expansion rate was 3.30 km^2^ y^−1^ and there were about 52.81 km^2^ changed to urban land during 1974–1990. The urban extension rate (4.48 km^2^ y^−1^) in the next ten years of 1990–2000 was obviously higher than that in the initial stage. Since 2000, the urban expansion started with a sharp speed of 10.28 km^2^ y^−1^ which was more than two times of 1990–2009, because of the rapid economic development and insistent population growth in this stage. The urban extension area reached to 92.20 km^2^ within 10 years, and the values of urbanization intensity index were 0.12, 0.16 and 0.30 in stages of 1974–1990, 1990–2000, and 2000–2009 (Table 2), respectively. The high value of urbanization intensity index in 2000–2009 indicated that the urban expansion was great in this period.

The urbanization expansion and landscape pattern change were influenced by many factors, such as geographical location, population, economic policy, resources, and transportation [25, 40]. The coastal location advantages played an important role in urbanization expansion [8, 9]. The spatial distribution of urban land expanded along the coastline (Figure 2). Influenced by port economy, the distribution of urban land in the study area from 1974 to 1990 was formed with the spatial construction of “one center,” which was similar with the urbanization process of many coastal cities which depended on port economy in the world [26, 41]. In the studied area, The GDP increased from 4.69 billion YUAN in 1990 to 155.30 billion YUAN in 2009; the total population grew about 0.70 million from 1990 (1.41 million) to 2009, resulting in about 137 km^2^ urban land increase (Table 2) and about 237 km^2^ farmland lost during this period (Table 3). The fast speed urbanization expansion started since 2000. From 2000 to 2009, the area of urban land added 92.20 km^2^ and urbanization intensity index was 0.38. Meanwhile, the urban space had transformed to axial expansion that developed from compact nutty to dispersed groups (Figure 2), which was similar with the axial expansion of Shanghai city between 1979 and 2000 [25]. Furthermore, with the ideology changes of urban construction policy [42] and transportation construction [25], the urban land of Yantai city has expanded to inner regions with the T-shape along the coastline (Figure 2), then has gradually aggregated from dispersed. In recent years, the agglomeration growth pattern of urbanization expansion became more and more obvious.

### 3.2. Analysis of Land Use/Cover and Landscape Pattern Changes

The dominant land use in study area in 1990 was farmland (48.45%), forestland (24.17%), and grassland (7.91%), the sum of which was more than 80% of the total area (Table 3). The urban land covered only 82.24 km^2^ (Figure 3), no more than 3% of the total area. In 2000, farmland and grassland had reduced to 1173.11 km^2^ and 163.72 km^2^, respectively, while the areas of urban land, orchard, forestland, water body, and other construction land increased greatly. Compared to 1990, the orchard, urban land, and forestland increased by 92.44 km^2^, 44.76 km^2^, and 40.03 km^2^, respectively (Table 3). A similar pattern of land use/cover change trend was found in 2009. Farmland continued shrinking to 1081.05 km^2^ and grassland decreased to 25.21 km^2^ from 2000 to 2009. The fastest growth of land uses was urban land instead of orchard with an increase of 92.20 km^2^ within 10 years. Many studies reported that the acceleration of urbanization expansion was the main driving force for decrease of farmland and green land [14, 31, 40, 43]. Farmland was the largest land use type in the study area (Table 3). During the period 1990–2009, the area of urban land increased greatly (about 137 km^2^) and farmland decreased from 1318.33 km^2^ in 1990 to 1081.05 km^2^ in 2009 (Figure 3 and Table 3). The result was similar with another Chinese coastal city, Longkou city [31]. Due to development of fruit economy, part of farmland was transformed into orchard (Table 3). The area of forestland increased from 1990 to 2000 because of the implementation of “returning farmland to forestland and grassland” project, while decreasing from 2000 to 2009 due to human activities increase and ecology degradation. In contrast, the forestland, water body, and other object land tented to decrease and most of lost areas had been transformed into maritime industrial land and tourism land.

The calculation results of landscape metrics which indicate landscape pattern change showed that the LPI decreased from 23.32 in 1990 to 20.35 in 2009 (Table 4), indicating that the dominant landscape of farmland had declined. Due to the strengthen of human interferences, the study area showed more fragmented landscapes, fewer large patches, which caused the Dominance and CONTAG decrease and FD increase during 1990–2009 (Table 4). Meanwhile, the urban land rapidly expanded from the urban center to the surrounding areas and then aggregated to a new large patch. The increase of SHDI and SHEI from 1990 to 2009 indicated that the landscape shapes in the study area had become more and more complex because of human activities [8].

### 3.3. Analysis of Regional Ecological Risk

**Figure 4:**
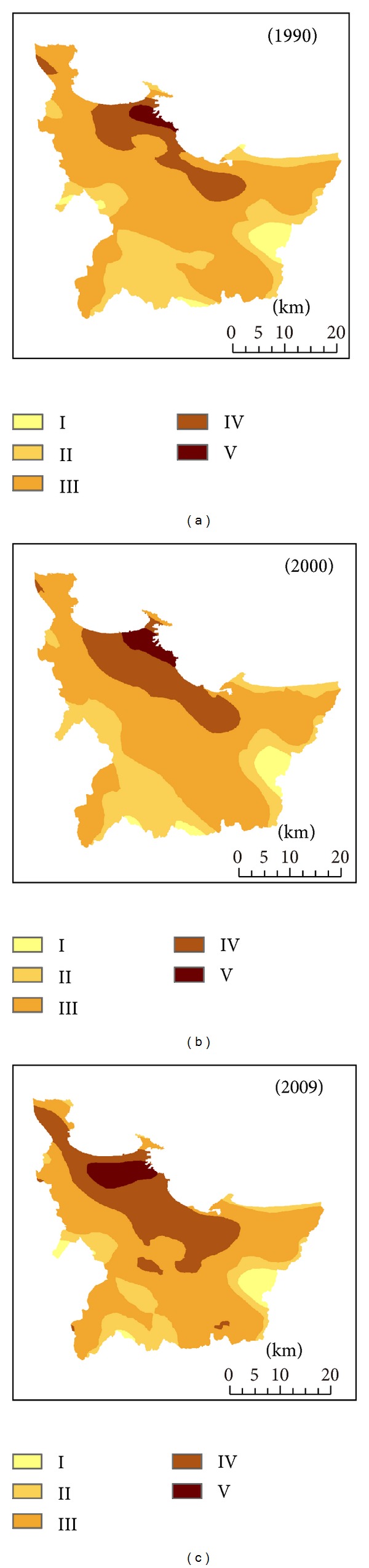
Spatial distribution of ecological risk in 1990–2009 ((I) low ecological risk, (II) sublow ecological risk, (III) moderate ecological risk, (IV) subhigh ecological risk, and (V) high ecological risk).

**Figure 5:**
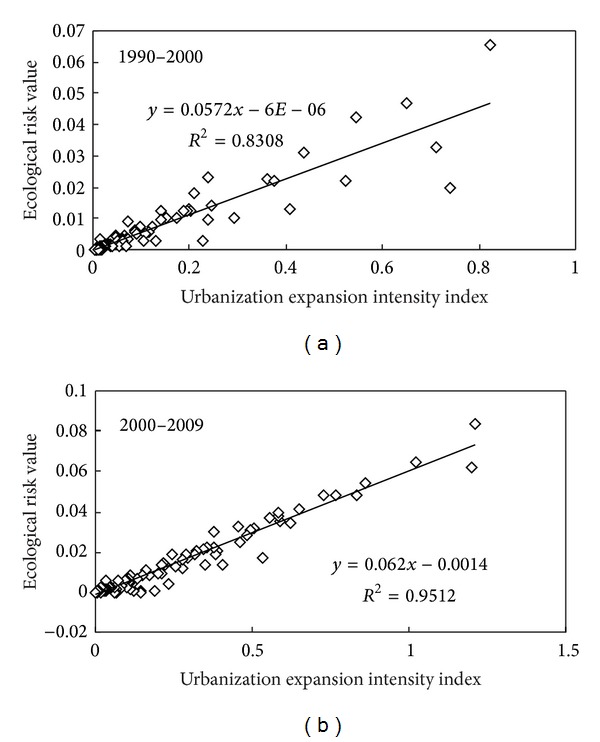
The relationship between urbanization intensity index and region ecological risk.

Using the Natural Breaks classification method [21], the ecological risk was grouped into five grades, that is, low ecological risk (I), sublow ecological risk (II), moderate ecological risk (III), subhigh ecological risk, and (IV) and high ecological risk (V), according to the ecological risk index. The results showed that the high and subhigh ecological risk areas are mainly distributed in the urban land, while low and sublow ecological risk areas appeared in forestland and grassland with high vegetation coverage (Figure 4). In addition, the ecological risk level decreased with the distance to the coastline increase. From 1990 to 2009, the moderate ecological risk level was major, and the proportions of subhigh and sublow ecological risk changed obviously in study area (Figure 4 and Table 5). The areas of moderate and sublow ecological risk decreased to 145.12 km^2^ and 266.47 km^2^ from 1990 to 2009, respectively. The low ecological risk area reduced to 4.5 km^2^. On the contrary, the area of high ecological risk increased from 37.79 km^2^ in 1990 to 61.47 km^2^ in 2000 and expanded rapidly to 104.18 km^2^ in 2009. The high ecological risk region mainly distributed along the coastline of Zhifu District and expanded westward to Fushan District and Development Zone (Figure 4). The area of subhigh ecological risk in 2009 was 2 times higher than that in 1990 and expanded to inner region from inshore region (Table 5 and Figure 4). The ecological security in the coastal zone was largely influenced by urbanization expansion and land use change [8, 16, 32, 44]. As the sensitive area in land-ocean interactions zone, Yantai city is located in the integrated high ecological risk area of the Ring-Bohai Region [16, 45]. In this study, the area of high and subhigh ecological risk region increased rapidly and extended eastward and westward along the coastline with layered distribution and the area of moderate and sublow ecological risk region reduced under the development of urbanization expansion (Figure 4). Such changes, especially the increasing trend of high ecological risk, were also observed in the coastal economic developing zone of Jinzhou Bay in Liaoning Province and Yancheng coastal area in Jiangsu Province [29, 45]. Previous studies predicted that more and more farmland and coastal water of Yantai city will be lost in the next 40 years due to the acceleration of urbanization process; a series of coastal development activities and reclamation projects would enhance the regional ecological risk [32]. The significant positive linear relationship between the urbanization intensity index and regional ecological risk index both in 1990–2000 and 2000–2009 with the determination coefficient (*R* ^2^) of 0.831 and 0.951, respectively, (Figure 5) was observed in the study, which obviously supported these prediction results. The results of this study suggested that the scientific urban development plan and rational coastal management measures should be implemented in order to reduce the ecological risk brought from urbanization expansion.

## 4. Conclusions

With economic development and population growth, the process of urbanization of Yantai city had accelerated obviously and the area of urban land increased quickly. The area of urban land increased about 137 km^2^ and about 237 km^2^ farmland lost during the period of 1990–2009. The distribution of urban land showed a T-shape along the coastline extent to inner regions. The area of high ecological risk increased rapidly and the moderate and sublow ecological risk decreased from 1990 to 2009. The high and subhigh ecological risk areas mainly distributed in the urban land and the ecological risk level decreased with the distance to the coastline increase. The significant positive relation between urbanization intensity and regional ecological risk was found in the study.

## Figures and Tables

**Table 1:** Description of landscape pattern metrics.

Index	Equation		Ecological significance
Largest Patch Index (LPI)	$\text{LPI}=\frac{\max a_{ij}}{A}\left(100\right)$	(i)	To indicate ratio of the largest patch area to total landscape area
Fractal Dimension Index (FD)	$\text{FD}=\frac{2\ln\left(l/k\right)}{\ln\left(A\right)}$	(ii)	To reflect the complexity of self-similarity of a patch
Shannon's Diversity Index (SHDI)	$H=-\overset{m}{\underset{i=1}{\sum }}\left(p_{i}\ln p_{i}\right)$	(iii)	To reflect landscape heterogeneity
Shannon's Evenness Index (SHEI)	$E=-\overset{m}{\underset{i=1}{\sum }}\frac{\left(p_{i}\ln p_{i}\right)}{\ln m}$	(iv)	To indicate even degree of different landscape types
Dominance Index (Dominance)	$\text{Do}=\ln m+\overset{m}{\underset{i=1}{\sum }}\left(p_{i}\ln p_{i}\right)$	(v)	To what extent several principal landscape types control whole landscape
Contagion Index (CONTAG)	$\text{CONTAG}=\left[1+\frac{\sum_{h=1}^{m}\sum_{i=1}^{m}\left[\left(p_{i}\right)(g_{hi}/\sum_{i=1}^{m}g_{hi})\right]\left[\ln\left(p_{i}\right)(g_{hi}/\sum_{i=1}^{m}g_{hi})\right]}{2\ln\left(m\right)}\right]\left(100\right)$	(vi)	To express the agglomeration degree among different landscape types

*I*:patch type; *j*: number of patch; *A*: the total area; *a*_*ij*_: the area of patch *ij*; *l*: patch perimeter; *k*: a constant, as to grid map, *k* equals to 4; *g*_*hi*_: contiguity number among patch *h* and patch *i*; *p*_*i*_: the area ratio of class *i*; *m*: quantity of region landscape types.

**Table 2:** Urbanization extension in Yantai city in different historical periods.

Different period	Extension area (km^2^)	Annual expansion area (km^2^ y^−1^)	Urbanization intensity index
1974–1990	52.81	3.30	0.12
1990–2000	44.76	4.48	0.16
2000–2009	92.20	10.24	0.38
1974–2009	189.77	5.42	0.20

**Table 3:** Area and percentage of land use types in Yantai city from 1990 to 2009.

LUCC type	1990		2000		2009	
	Area (km^2^)	Percentage (%)	Area (km^2^)	Percentage (%)	Area (km^2^)	Percentage (%)
Urban land	82.24	3.02	127.00	4.67	219.20	8.06
Farmland	1318.33	48.45	1173.11	43.11	1081.05	39.73
Orchard	182.76	6.72	275.20	10.11	308.15	11.32
Forestland	657.62	24.17	697.65	25.64	662.41	24.34
Grassland	215.11	7.91	163.72	6.02	138.51	5.09
Water body	79.72	2.93	89.21	3.28	87.80	3.23
Other construction land	131.34	4.83	150.05	5.51	180.65	6.64
Other object land	53.92	1.98	45.10	1.66	43.27	1.59

**Table 4:** Dynamic change of landscape metrics from 1990 to 2009.

Year	LPI	FD	SHDI	SHEI	Dominance	CONTAG
1990	23.32	1.0671	1.55	0.71	0.29	54.49
2000	22.20	1.0679	1.62	0.74	0.26	52.72
2009	20.35	1.0682	1.68	0.76	0.24	51.26

**Table 5:** Area of different level ecological risk from 1990 to 2009.

Ecological risk grade	1990	2000	2009	1990	2000	2009
	Area (km^2^)			Area proportion (%)		
Low ecological risk region	92.97	91.47	88.47	3.42	3.36	3.25
Sublow ecological risk region	654.59	629.28	388.12	24.07	23.14	14.27
Moderate ecological risk region	1633.04	1550.90	1487.92	60.05	57.03	54.72
Subhigh ecological risk region	300.94	386.22	650.65	11.07	14.2	23.93
High ecological risk region	37.79	61.47	104.18	1.39	2.26	3.83
